# Wound swab quality grading is dependent on Gram smear screening approach

**DOI:** 10.1038/s41598-023-29832-1

**Published:** 2023-02-23

**Authors:** Shawn T. Clark, Jessica D. Forbes, Larissa M. Matukas

**Affiliations:** 1 Department of Laboratory Medicine and Pathobiology, University of Toronto, Toronto, Canada; 2 Department of Laboratory Medicine, St Michael’s Hospital, Unity Health Toronto, Toronto, Canada; 3 Present Address: Eastern Ontario Regional Laboratory Association, Ottawa, Canada

**Keywords:** Clinical microbiology, Infectious-disease diagnostics

## Abstract

Superficial skin swab collections are inherently low-quality and may be of little clinical value due to their poor sensitivity and specificity. Clinical microbiology laboratories can use Gram smears to screen and differentiate higher and lower quality specimens to direct the extent of potential pathogen work up, including antimicrobial susceptibility testing (AST). We compared the impact of two different smear grading approaches to our current reporting practices for superficial wound swab cultures. Two variations of the Q score methodology (low power under 10X (QS10) and high power under 100X (QS100) were compared to our existing oil immersion method (OM100) (100X). We further evaluated the QS100 method by scoring superficial swab smears previously screened by OM100 from cultures submitted between November 2018 and December 2019. No significant difference in the number of low-quality specimens (N = 50) was identified by QS10 or QS100 grading (N = 9; 18%; N = 8; 16% respectively). Among 968 additional QS100 screened smears, 67 (6.9%) low quality swabs were identified and 7.4% fewer organisms (76/1020 organisms) would require reporting with AST. Implementing the Q score for superficial wound swab cultures would provide minimal improvements in their clinical relevance, laboratory quality and efficiency in our laboratory due to the low number of poor-quality swabs received.

## Introduction

The use of swab collections to identify the causative agents involved in wound infections is a controversial topic among healthcare professionals^1–3^. This is in part because the microbiological analysis of wound cultures relies heavily on the quality of the specimen submitted to the laboratory. Whilst being relatively easy to perform, the pre-analytical aspects of wound specimen collection are highly variable. Common pre-analytical issues that compromise the quality of these specimens and complicate the reporting process include the lack of a consistent swab collection method within an institution, the absence of specific clinical indication for collection of a specimen for wounds (e.g. wound is non-purulent, inflammation is limited and/or wound is healed), inadequate descriptions of the anatomical site sampled provided to the laboratory, among others^4–6^.

When used in combination with known clinical signs and symptoms of infection (e.g. pain, erythema, pus, foul odour and chronicity, among others), superficial wound swab cultures can assist providers with identifying pathogens and thus selecting an appropriate therapy^3^. Specimens that are contaminated with the commensal microbiota likely offer little clinical benefit as traditional pathogens may be overgrown by less significant organisms^4^. Microbiology laboratories may process superficial wound swabs by examining a Gram smear, evaluating the culture and performing antimicrobial susceptibility testing (AST) where necessary^6^. The Gram smear is a critical tool in evaluating specimen quality by reporting on the relative abundance of squamous epithelial cells (SEC) compared to polymorphonuclear (PMN) cells. The quality of the specimen, based on Gram stain scoring, can then be used to guide further work up to distinguish superficial contaminating microorganisms from infectious ones. Whilst guidance exists for applying Gram stain grading systems to wound swab specimen quality^6–8^, these approaches have not been universally adopted and are not part of the standard of care for superficial wound infections around the world^8–12^.

The Q score system is a stringent quality grading approach that can guide both analytical and post-analytical processes, such as organism identification (ID), AST and their reporting, by using SECs and PMNs as indicators of specimen quality^7,8^. In this model, numerical values are assigned to SEC (0 to − 3) and PMNs (0 to 3) and scores are translated into low (Q_0_), moderate (Q_1_, Q_2_) or high quality (Q_3_) categories based on the possible permutations of SEC and PMN in a decision matrix^8^. Here, the Q score defines the number of potential pathogens (e.g. *Staphylococcus aureus*) that would be reported with ID and AST for a given specimen, with a maximum of three pathogens being reported from a Q_3_ specimen. With this approach, laboratories could adopt a Q_0_ score as a threshold for acceptability of a swab for culture, however some clinically significant pathogens may be missed in these specimens. There is little data available to indicate the potential impact of a change in reporting on laboratory utilization or clinical practice^8–10^.

The objective of our study was to evaluate the impact of Gram smear grading on the characterization and reporting of superficial wound swab cultures. We compared three approaches to evaluate swab collection quality through SEC and PMN abundance: (i) the current method used by the laboratory which reports SEC and PMNs semi-quantitatively after screening under high power (100X) (OM100)^11^ and the Q score grading under (ii) low power (10X) (QS10)^8^ and (iii) high power (100X) (QS100). We applied QS10 and QS100 retrospectively to previously processed and reported wound swab cultures to determine whether a change in screening approach impacted the analytical and post-analytical processing of superficial swabs in a tertiary care hospital microbiology laboratory.

## Methods

### Description of current laboratory swab culture workup

Superficial swabs are collected and submitted to the microbiology laboratory at St. Michael’s Hospital, Unity Health Toronto (Toronto, Canada) in eSwab transport media (COPAN, Italy). This institution recommends swab collection be performed using the Levine technique^5^. Submitted swabs are processed for a direct Gram smear and routine bacterial culture as per the laboratory’s standard operating procedures. As part of the microscopic examination (OM100), the laboratory reports SEC and PMN semi-quantitatively (occasional, few, moderate, many) in accordance with a method described by Church et al.^11^ (Table 1). These screens are performed under high power (100X lens) with oil. Here, the Gram smear result does not preclude any culture workup and is provided as a quality metric that clinicians can use to interpret the culture result.

**Table 1:** Criteria used to evaluate Gram smears of wound swabs submitted for culture.

Current laboratory grading scheme (OM100)^a^		Q score grading scheme (QS10 and QS100)^b^		
# of cells per field (100X)	Score (PMNs and SECs)	# of cells per field (10X or 100X)^c,d^	Score (PMNs)	Score (SECs)
< 1	Occasional	0	0	0
1–5	Few	1–9	1	− 1
5–10	Moderate	10–24	2	− 2
≥ 10	Many	≥ 25	3	− 3

^a^OM100 represents the Gram smear grading method used by the laboratory at the time of the study which provides a semi-quantitative assessment of the presence of squamous epithelial cells (SECs) and polymorphonuclear cells (PMNs) under high power (100X objective).

^b^The Q score was determined by assessing the number of PMNs and SECs per field under (ii) low power (10X objective) (QS10) and (iii) high power (100X objective) (QS100).

^c^The same number of cells per field were used for scoring under both QS10 and QS100.

^d^The number of cells per field (100X) from the OM100 scheme was used for the QS100 Q score criteria applied to retrospectively collected wound swabs.

Organisms identified in culture are then classified as primary pathogens (e.g. *S. aureus, S. pyogenes, P. aeruginosa*), possible pathogens (e.g. enterococci) or commensal microbiota (e.g. skin organisms) based on their MALDI-TOF MS ID, abundance on primary culture plates and the anatomical site of collection (e.g. sterile or non-sterile); these classifications were defined according to the Clinical Microbiology Procedures Handbook^6^. ID and AST is performed on (i) a primary pathogen (any amount), (ii) pure growth of a potential pathogen (any amount), or (iii) up to 3 potential pathogens from the same specimen. Minimal testing is performed on potential pathogens (ID only) when moderate or many SECs are present in the Gram smear, or if no evidence of infection is noted (e.g. no PMNs or clinical information provided to indicate an infection).

### Comparison of grading criteria for superficial wound swab Gram smears

This initiative was formally reviewed by institutional authorities at Unity Health Toronto and deemed to require neither Research Ethics Board approval nor written consent from participants. All methods were carried out in accordance with laboratory procedures and institutional policies. The OM100, QS10, and QS100 comparison was performed on smears from randomly selected superficial swabs submitted to the microbiology laboratory between January 8th 2020 and February 20th 2020. Gram smears were previously examined by the OM100 method and were prospectively re-graded by standard Q score criteria under low power (QS10)^8^, or a third method where Q scores were assigned under high power magnification with oil (QS100) (Table 1). Microscopic examinations were performed using a Nikon Eclipse 50i (Nikon Corporation, Japan), with a minimum of ten fields being scanned per slide by a single reader under either magnification. Numerical values were assigned to SECs and PMNs as described in Table 1 and an overall score determined as per Matkoski et al.^8^. A score of 0 (Q_0_) indicates a low-quality swab collection, 1 (Q_1_) and 2 (Q_2_) represent moderate quality and 3 (Q_3_) represents high quality. In this study, a Q score was used only for research purposes.

### In silico validation of a Q score approach with retrospective swab culture data (IS-QS)

To evaluate the potential impact that changing to a Q score quality metric may have on swab culture workup in our laboratory, we performed an in silico Q score exam (IS-QS) using retrospectively collected culture data. Here, QS100 criteria were applied retrospectively (Table 1) to OM100 reports for all superficial wound swabs submitted to the laboratory between November 29th, 2018 and December 23rd, 2019 and a predicted Q score was generated. We simulated culture workup as described previously^8^, with up to three pathogens (primary or possible) being processed for ID and AST from Q_1_, Q_2_ or Q_3_ specimens respectively and none from Q_0_ specimens. In situations where the number of pathogens was greater than the Q score, the Gram smear determined whether both ID and AST or ID alone would be warranted (e.g. ID and AST for all potential pathogens only if Gram and culture were concordant). We defined the impact of the in silico Q score as a difference in the number of swabs processed, the number of organisms with ID (primary, potential or commensal) and any AST performed. To identify whether particular submitters within the hospital network were more likely to collect poor quality specimens, we stratified this data by the location of specimen collection (e.g. outpatient clinic or specific hospital ward). Patient clinical data including patient outcomes and treatment data were not included in this study.

### Data analysis

Study variables including the proportion of swab types examined during the study period, Q score comparisons and the proportion of poor quality (Q_0_) specimens were summarized using descriptive measures. Comparisons in swab quality grading between QS10 and QS100 and differences in the number of isolates processed for ID and/or AST were performed using the Fisher’s exact or Chi square test where appropriate in GraphPad Prism v 9.1.1. (GraphPad Software LLC, USA).

## Results

### Comparison of wound swab quality by OM100, QS10, QS100 methods

**Figure 1:**
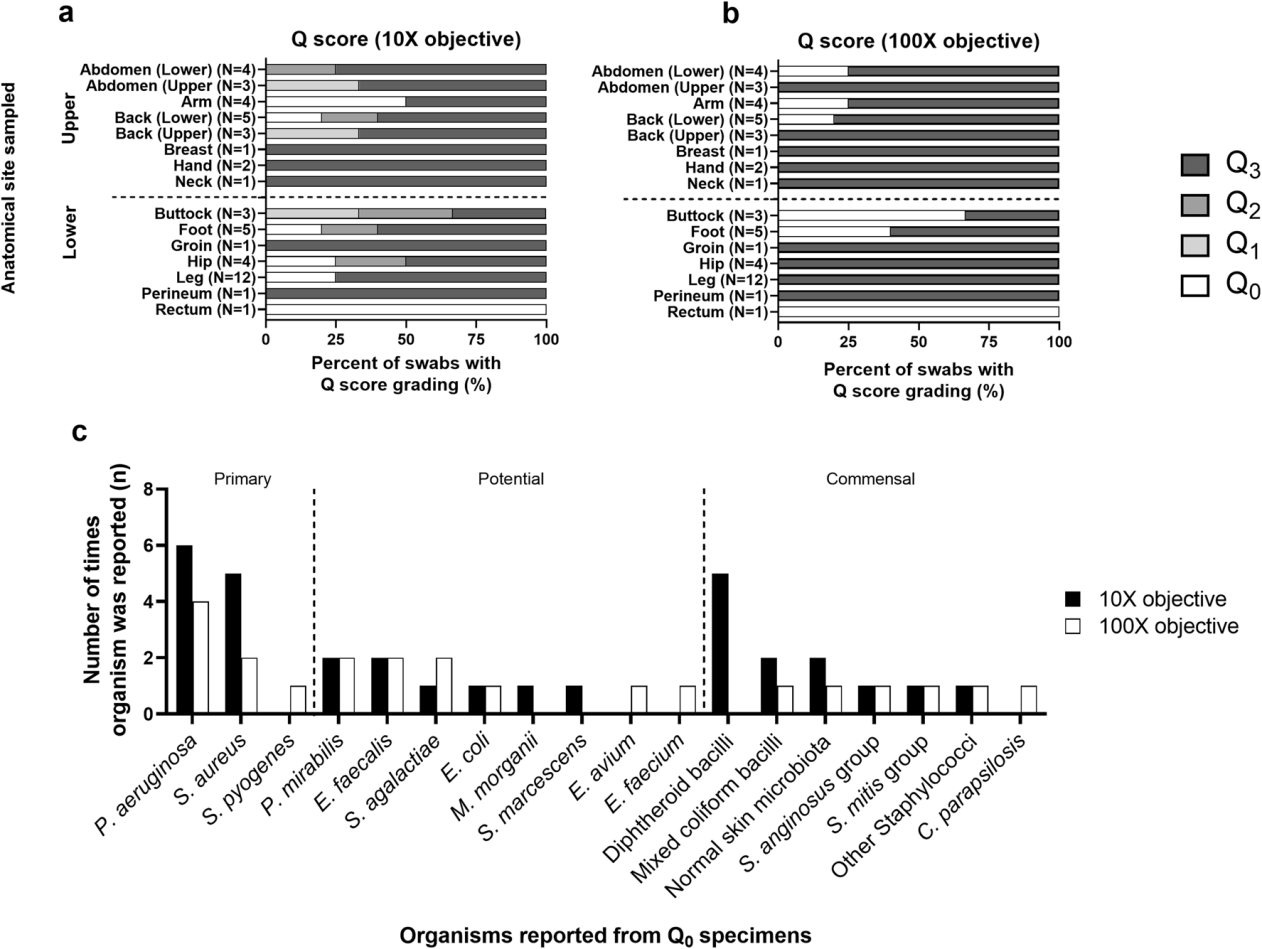
Comparison of Gram smear grading approaches to evaluate wound swab quality. Swabs (N = 50) from 40 patients were examined using Q score grading under (**a**) low power (10X) and (**b**) high power (100X) objectives to mimic the criteria currently used by the laboratory (100X semi quantitative reporting of PMN, SEC and organisms) and (**c**) differences in pathogens identified among Q_0_ swabs. Organisms were classified as primary or potential pathogens or commensal organisms was based on current laboratory protocols.

The OM100, QS10 and QS100 were compared using 50 randomly selected superficial swab Gram smears from 40 patients (Fig. 1). Given the type of grading schemes employed, differences in the laboratory interpretation of swab quality were only quantifiable between QS10 (Fig. 1a) and QS100 (Fig. 1b) grading approaches. OM100 requires a clinical interpretation of quality, as the microbiology laboratory does not currently reject low-quality swabs (Fig. 1; Table S1). A greater number of Q_3_ specimens were identified by QS100 (N = 42; 84%) and less variability in grading was noted, as scores of only Q_0_ or Q_3_ were reported. In contrast, greater variability in swab quality was found with QS10, as 16% were classified into moderate quality (Q_1_ or Q_2_) categories. There was however no significant difference in the proportion of low (Q_0_) or acceptable quality (Q_1_, Q_2_, Q_3_) specimens reported under QS10 or QS100 (N = 9; 18% vs. N = 8; 16%, respectively; *P* = 0.59) (Fig. 1). Only four smears were assigned a Q_0_ score under both QS10 and QS100. Thirteen swabs (26.0%) were discrepant between QS10 and QS100 grading (Table S1). Five swabs (33.3%) changed from Q_0_ to Q_3_ between QS10 and QS100 respectively and four (31.0%) were graded as Q_0_ by QS100 despite being of moderate quality by QS10. Overall, the majority of swabs would be acceptable by both QS10 (82.0%, N = 41) or QS100 (84.0%, N = 42) grading. We noted however that a change in grading to QS10 would identify more low-quality swabs with commensal microbiota (e.g. diphtheroid bacilli, mixed coliform bacilli and normal skin microbiota) and some primary pathogens by culture when compared with QS100 (Fig. 1c).

### Validation of in silico Q score with retrospectively collected clinical specimens (IS-QS)

**Figure 2:**
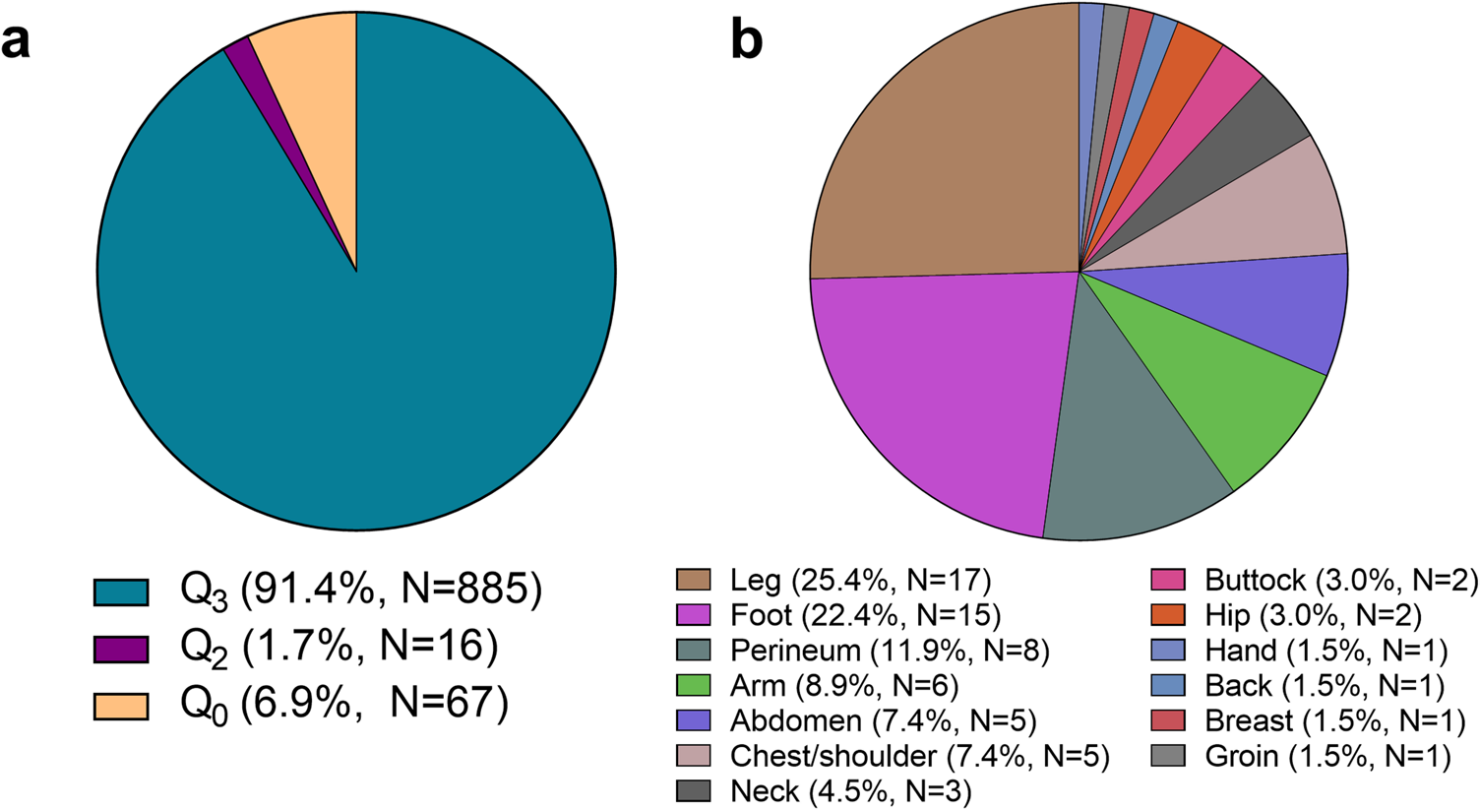
Summary of Q score validation with retrospectively analyzed data (IS-QS). Specimens graded in silico by the QS100 scheme (high power) (N = 968) were examined for (**a**) predicted overall quality (Q_1_ not displayed, N = 0), and the corresponding Q_0_ swabs identified in the IS-QS set further classified by (**b**) proportion anatomical site of collection (N = 67).

The IS-QS validation set consisted of 968 wound swabs that were submitted for OM100 Gram smear and routine culture over 390 days. Re-screening these smears in silico using QS100 criteria identified 885 Q_3_ swabs (91.4%) that would be acceptable for culture (Fig. 2a). Specimens of moderate quality (Q_1_ and Q_2_) accounted for 16 (1.7%) swabs, while 67 (6.9%) were considered low quality (Q_0_). The Q_0_ swabs (N = 67) among the IS-QS set were more frequently from the leg (N = 17; 25.4%), foot (N = 15; 22.4%) and perineum (N = 8, 11.9%). Due to the inherent differences in OM100 and QS100 grading schemes (Table 1), statistical comparison was not possible. (Fig. 2b).

**Figure 3:**
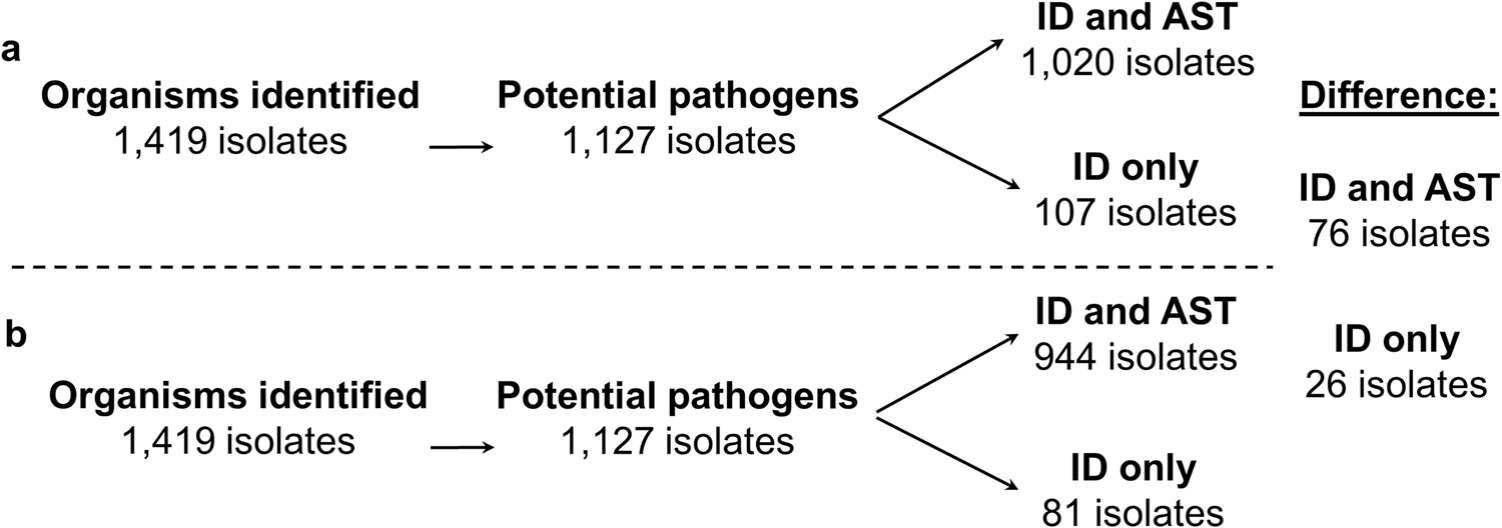
Comparison of wound swab reporting between (**a**) the existing OM100 semi-quantitative grading and pathogen reporting scheme and after (**b**) Q score criteria has been applied. This analysis includes the retrospective sample set with the OM100 score translated into the Q100 score.

When compared to the OM100 method for IS-QS cultures, we noted a difference in the workup of 102 bacterial isolates that would receive either both ID and AST, or ID alone, following screening by QS100 criteria (Fig. 3). Among the OM100 screen, a total of 1020 organisms from the IS-QS cultures received both ID and AST (Fig. 3, Table S2). Of the remaining organisms, 107 received ID only and AST results were referred to a previous specimen from the same patient (collected within 3 or 7 days for Gram-negative and Gram-positive organisms, respectively) for 85 isolates as per current laboratory procedures. With the in silico QS100 quality predictions and the implication of this grading criteria for laboratory analytical processes, both ID and AST would be recommended for 944 organisms from IS-QS swabs, while only 81 would receive ID alone. While the difference in the number of isolates processed for ID and/or AST by either method was not statistically significant (*P* = 0.19), 84 potential pathogens were isolated from Q_0_ specimens (N = 67), including *S. aureus* (N = 26 methicillin sensitive, N = 2 methicillin resistant), *E. faecalis* (N = 8), *S. pyogenes* (N = 8) and *S. agalactiae* (N = 7) (Table S2), which may not be reported with the adoption of a Q score-driven grading scheme.

### Identification of submitters of low-quality swabs in the healthcare facility

**Figure 4:**
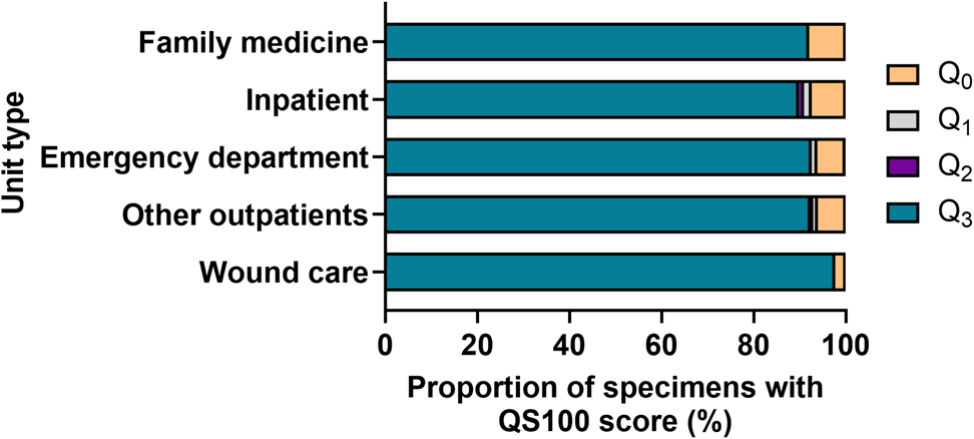
Examination of swab quality across different submitters within the hospital network. The number of in silico predicted Q_0_ specimens submitted over the 390 day period was examined by units submitting swabs for routine culture. Submitters were classified as inpatient (including plastic surgery, general medicine, cardiovascular surgery, cardiology, among others) (N = 33/444, 7.43%), family medicine (N = 16/201, 7.96%), other outpatients (including hemodialysis, fracture clinic, gynecology, among others) (N = 12/212, 6.06%), emergency department (N = 5/81, 6.17%) or wound care (N = 1/44, 2.27%).

To identify the origins of low-quality swabs within the healthcare facility, we examined submitter information for all Q_0_ swabs (e.g. patient units and/or clinics; Fig. 4). Units submitting swabs were categorized as inpatient (N = 444; 45.6%), family medicine (N = 201; 20.8%), other outpatients (N = 212; 20.5%), emergency department (N = 81; 83.7%) and wound care (N = 44; 4.5%). Family medicine clinics (7.96%) and inpatient units (7.43%) were found to submit the highest percentages of Q_0_ swabs (Fig. 4) at our institution.

## Discussion

Swab cultures are challenging for microbiology laboratories to process, as interpreting the significance of bacterial growth is highly dependent on sample quality. In addition, Gram smears are not universally considered standard of care around the world. For instance, a Gram smear is not required by Public Health England guidance for superficial swab cultures in the United Kingdom^12^, and a 2010 survey of the patterns of practice among laboratories in Ontario, Canada identified that most laboratories did not use Gram smear for screening^13^. However, recent recommendations from Choosing Wisely Canada^14^ and guidance on swab culture workup described by Leber and colleagues^6^ suggest that Gram smears are critical components of superficial swab examination. In this study we noted that the Gram smear approach used to assess wound swab quality could influence the classification of specimens as high, moderate or low quality by the Q score system and may lead to some primary pathogens being missed in critical specimens if low quality swabs are withheld from routine culture.

We determined that the quality grading of wound swab Gram smears based on Q score criteria differed under high and low power magnification in a subset of swabs submitted for routine culture. Standard Q score grading, as described by Bartlett et al.^7^ for respiratory specimen smears and Matkoski et al.^8^ for wound swab smears, is traditionally performed under low power magnification. To minimize overall change to our current laboratory practices, we chose to assess smear grading under both high and low power, as high power/oil screens^11^ are in use in our laboratory. Less variability in quality grading by Q score was noted among our subset of wound swabs when screened by high power fields. With this approach, fewer specimens were deemed low quality and primary pathogens identified in these cultures would continue to be reported with identification and susceptibilities where appropriate. In contrast, we noted greater variability in quality with low power screening which if these specimens were rejected by smear or withheld for culture workup, would result in important primary pathogens such as *S. aureus* not being identified.

In keeping with patterns observed among the randomly sampled swabs included in the Gram smear comparison set, we noted that few Q_0_ specimens were predicted among 968 wound swabs collected over a 390-day period, when evaluated in silico. Both ID and AST would be predicted/recommended for 944 organisms, while an additional 81 would receive partial workup (identification only) based on smear quality. This represented a relatively minor difference in workup for 76 (full) and 26 (partial) isolates, respectively when compared to current laboratory approaches. Our results are contrasting with similar studies^8–10^ which noted a significant difference in the number of wound swab specimens that would be or were interpreted differently using the Q score (e.g. a greater number of low quality specimens). It is difficult to compare these studies for several reasons. The previous studies had used standard Q score grading using low power magnification (versus our studies high power magnification) and secondly, our institution had previously initiated quality improvement practices related to swab collection and therefore our swab quality was relatively good in comparison. Additional studies across various healthcare facilities would be beneficial to further assess the potential impact of this procedural change on laboratory testing volumes and to identify units where further intervention may be needed (e.g. diagnostic and antimicrobial stewardship, education on specimen collection and others).

It is important to note that using the Q score alone to reject low quality specimens may result in some primary pathogens being missed. Laboratories that are considering implementing such screening approaches should evaluate their specimen retention policies, and communicate a time period within which the primary care team can contact the laboratory to request workup on a Q_0_ specimen. The low number of predicted Q_0_ swabs at our facility may be due to prior quality improvement interventions over the past decade that involved repeated education on the poor quality of wound swabs and promotion of collecting specimens with higher diagnostic yield. In this setting, implementing Q score grading will have minimal quality improvement due to the small percentage of Q_0_ swabs (reassuring of our collection practices).

Our study had several limitations. Sample size was a limiting factor for our initial smear comparison study and only a limited analysis could be performed. We did not assess the potential implications of a change in smear grading on antimicrobial stewardship practices and clinical management and are thus unable to quantify the impact of this change outside of the laboratory setting. Similarly, we recognize that methods of superficial swab collection may not have been standardized at the point of collection, however, the Levine technique has been promoted in wound care education throughout the institution. We are also unable to extrapolate these findings to swabs collected specifically for the culture of obligate anaerobic bacterial pathogens. Additional studies are needed to further investigate these factors.

It is important to note that laboratory grading schemes are not the sole solution to the quality issue surrounding wound swab cultures. While the techniques examined in this study may represent our best efforts to reduce the number of swabs processed by the microbiology laboratory, the result may not always be clinically actionable. Improving the overall quality of swab cultures and laboratory utilization requires an interdisciplinary intervention that involves communication and collaboration among healthcare providers and may include interventions such as the implementation of continuing education programs related to swab collection and the indications for their collection. For laboratories that are interested in adopting a Q score approach as part of quality improvement and diagnostic stewardship initiatives, our study suggests the influence of microscopic technique on the interpretation of quality indicators should also be considered.

## Conclusions

The use of a Q score grading approach for wound swabs had minimal potential impact on downstream workflow in our laboratory. Laboratories should consider introducing the Q score to improve the efficiency and quality of wound swab cultures. The adoption of this method may be most useful for laboratories that serve healthcare facilities that do not have rigorous education on collection practices and where additional quality improvement measures have not been previously introduced. Additional studies are required to determine the significance of introducing limitations to Q_0_ specimen workup and its potential impact on patient management.

## Supplementary Information

Supplementary Tables.

## Data Availability

All data generated or analyzed during this study are included in this published article and its supplementary information files.
