# Supplementary material for: Wound swab quality grading is dependent on Gram smear screening approach
Source: Sci Rep. 2023 Feb 23;13:3160. doi: 10.1038/s41598-023-29832-1 (PMC9950449; doi:10.1038/s41598-023-29832-1)
Supplement: Supplementary file 1 — Supplementary Tables. [file 41598_2023_29832_MOESM1_ESM.pdf]

Supplementary Material

for

Wound swab quality grading is dependent on Gram smear screening approach

Shawn T. Clark[1*], Jessica D. Forbes[1,2,*], Larissa M. Matukas[1,3,#]

This file includes:

Table S1

Table S2

Table S1. Comparison of microscopic grading of wound smears by OM100, QS10 and QS100

Anatomic site	Q score grading						Current laboratory grading scheme	
	Low power (10X) (QS10)			High power (100X) (QS100)			High power (100X) (OM100)	
	PMN	SEC	Q Score	PMN	SEC	Q Score	PMN	SEC
Abdomen (lower)	>24	0	3	10-24	0	3	Many	No
	0	0	3	0	0	3	No	No
	>24	0	3	>24	0	3	Many	No
	>24	1-9	2	1-9	1-9	0	Few	Occasional
Abdomen (upper)	>24	0	3	1-9	0	3	Moderate	Few
	10-24	0	3	1-9	0	3	Few	No
	10-24	1-9	1	1-9	0	3	Moderate	No
Arm	0	0	3	0	0	3	No	No
	1-9	0	3	1-9	0	3	Few	No
	1-9	1-9	0	1-9	0	3	Few	No
	1-9	1-9	0	1-9	1-9	0	Occasional	No
Back (lower)	1-9	1-9	0	1-9	1-9	0	Occasional	Occasional
	0	0	3	1-9	0	3	Occasional	Occasional
	0	0	3	0	0	3	No	No
	>24	1-9	2	10-24	0	3	Many	No
	1-9	0	3	1-9	0	3	Occasional	No
Back (upper)	0	0	3	0	0	3	No	No
	0	0	3	0	0	3	No	No
	1-9	10-24	1	0	1-9	3	Few	No
Breast	>24	0	3	>24	0	3	Many	No
Buttock	10-24	1-9	1	1-9	1-9	0	Moderate	Moderate
	>24	0	3	0	10-24	3	Occasional	No

	>24	1-9	2	1-9	1-9	0	Few	No
Foot	0	0	3	0	0	3	No	No
	0	1-9	0	0	1-9	0	Occasional	No
	>24	1-9	2	1-9	1-9	0	Moderate	No
	>24	0	3	10-24	0	3	No	No
	1-9	0	3	1-9	0	3	Few	No
Groin	0	0	3	0	0	3	No	No
Hand	0	0	3	0	0	3	No	No
	10-24	0	3	1-9	0	3	Many	No
Hip	1-9	1-9	0	1-9	0	3	Occasional	Occasional
	>24	1-9	2	1-9	0	3	Occasional	No
	0	0	3	0	0	3	No	No
	>24	0	3	>24	0	3	Moderate	Occasional
Leg	0	0	3	0	0	3	No	No
	0	0	3	0	0	3	No	No
	10-24	0	3	1-9	0	3	Occasional	No
	10-24	0	3	1-9	0	3	Occasional	No
	0	1-9	0	1-9	0	3	Few	No
	0	1-9	0	0	0	3	Occasional	No
	0	0	3	1-9	0	3	Few	No
	0	1-9	0	0	0	3	No	No
	0	0	3	0	0	3	No	Occasional
	10-24	0	3	1-9	0	3	No	No
	0	0	3	0	0	3	No	No
	0	0	3	1-9	0	3	No	No
Neck	0	0	3	0	0	3	No	No
Perineum	>24	0	3	10-24	0	3	Moderate	No

Rectum	0	1-9	0	0	1-9	0	Occasional	Occasional

Table S2. Organisms identified from IS-QS swab cultures graded as Q_0 by OS100

Q_0 swab	Organism 1 (Quantity)	Organism 2 (Quantity)	Organism 3 (Quantity)	Organism 4 (Quantity)	Other organisms (Quantity)
1	CNST (VL)	-	-	-	-
2	*S. aureus* (H)	-	-	-	-
3	Normal skin microbiota	-	-	-	-
4	*S. aureus* (H)	Normal skin microbiota (H)	Mixed coliform bacilli (L)	-	-
5	Actinomyces spp. (H)	Achromobacte r spp. (VL)	Normal skin microbiota (L)	-	-
6	Mixed coliform bacilli (H)	E. faecalis (H)	Normal skin microbiota (H)	-	-
7	S. aureus (L)	E. cloacae complex (VL)	S. anginosus group (L)	Diphtheroid bacilli (L)	-
8	E. cloacae complex (M)	E. faecalis (H)	S. aureus (VL)	Normal skin microbiota (VL)	-
9	No growth	-	-	-	-
10	Fecal flora	-	-	-	-
11	CNST (VL)	-	-	-	-
12	E. aerogenes (H)	-	-	-	-
13	S. aureus (H)	-	-	-	-
14	Group G streptococcus (H)	S. aureus (M)	Normal skin microbiota (H)	-	-
15	S. aureus (H)	S. agalactiae (H)	-	-	-
16	S. aureus (H)	S. agalactiae (H)	E. avium (H)	Normal skin microbiota (H)	-
17	P. mirabilis (M)	Group G streptococcus (H)	S. agalactiae (H)	Gemella spp. (M)	-
18	S. agalactiae	S. intermedius	M. morganii	-	-

	(H)	group (H)	(H)		
19	P. mirabilis (H)	E. faecalis (H)	S. aureus (VL)	Normal skin microbiota (VL)	-
20	S. aureus (L)	Normal skin microbiota (M)	-	-	-
21	S. pyogenes (H)	S. aureus (H)	Arcanobacteriu m haemolyticum (H)	-	-
22	S. aureus (H)	S. pyogenes (H)	S. maltophilia (VL)	Trichosporon spp. (L)	-
23	Proteus spp. (VL)	CNST (L)	-	-	-
24	S. aureus (H)	Group C streptococcus (H)	S. pyogenes (H)	MRSA (H)	Diphtheroid bacilli (L)
25	S. agalactiae (M)	Normal skin microbiota (L)	-	-	-
26	P. mirabilis (L)	Group C streptococcus (H)	Diphtheroid bacilli (H)	-	-
27	Mixed coliform bacilli (M)	Diphtheroid bacilli (H)	-	-	-
28	S. aureus (H)	Diphtheroid bacilli (H)	-	-	-
29	Normal skin microbiota	-	-	-	-
30	P. aeruginosa (M)	K. oxytoca (L)	S. aureus (L)	Normal skin microbiota	-
31	CNST (VL)	-	-	-	-
32	S. aureus (H)	Aerobic spore bearing bacilli (H)	-	-	-
33	S. dysgalactiae (H)	MRSA (H)	Proteus spp. (H)	S. pyogenes (H)	M. morganii (H) Alcaligenes faecalis (M) Arcanobacterium haemolyticum (H)

34	Normal skin microbiota	-	-	-	-
35	E. coli (L)	-	-	-	-
36	K. pneumoniae (M)	E. cloacae complex (M)	E. faecalis (M)	Normal skin microbiota (L)	-
37	P. aeruginosa (L)	S. aureus (VL)	-	-	-
38	S. agalactiae (M)	Normal skin microbiota (H)	-	-	-
39	S. aureus (H)	-	-	-	-
40	S. aureus (H)	S. agalactiae (H)	-	-	-
41	S. aureus (H)	-	-	-	-
42	Normal skin microbiota	-	-	-	-
43	CNST (VL)	-	-	-	-
44	S. aureus (H)	Group G streptococcus (H)	A. baumanii complex (L)	E. avium (L)	-
45	S. pyogenes (H)	S. aureus (M)	-	-	-
46	P. aeruginosa (M)	-	-	-	-
47	Normal skin microbiota	-	-	-	-
48	S. anginosus group (H)	K. pneumoniae (VL)	-	-	-
49	G. vaginalis (L)	Diphtheroid bacilli (VL)	-	-	-
50	S. aureus (M)	Alpha hemolytic streptococcus (M)	E. coli (H)	-	-
51	E. coli (H)	S. pyogenes (H)	Normal skin microbiota (L)	-	-
52	E. faecalis (H)	Lactobacillus	-	-	-

		spp. (M)			
53	Aerobic spore bearing bacilli (H)	Mixed coliform bacilli (H)	S. mitis group (H)	E. faecalis (L)	-
54	Mixed coliform bacilli (L)	S. anginosus group (H)	Diphtheroid bacilli (H)	E. faecalis (H)	-
55	E. coli (H)	Normal skin microbiota (H)	E. faecalis (H)	-	-
56	CNST (VL)	-	-	-	-
57	No growth	-	-	-	-
58	S. aureus (H)	Normal skin microbiota (H)	-	-	-
59	E. cloacae complex (L)	S. epidermidis (VL)	-	-	-
60	S. pyogenes (L)	CNST (VL)	-	-	-
61	Clostridium spp. (not perfringens) (VL)	Aerobic spore bearing bacilli (VL)	Actinomyces spp. (VL)	-	-
62	S. aureus (M)	Diphtheroid bacilli (M)	-	-	-
63	S. aureus (M)	Diphtheroid bacilli (M)	-	-	-
64	No growth	-	-	-	-
65	S. pyogenes (M)	Normal skin microbiota (L)	-	-	-
66	Normal skin microbiota	-	-	-	-
67	Normal skin microbiota	-	-	-	-

[a] Growth was quantitated as very light (VL), light (L), moderate (M), heavy (H) based on examination of the primary culture media
